# Detection of Morphine and Opioids in Fingernails: Immunohistochemical Analysis and Confirmation with Ultra-High-Performance Liquid Chromatography Coupled with High-Resolution Mass Spectrometry

**DOI:** 10.3390/toxics10080420

**Published:** 2022-07-26

**Authors:** Roberto Scendoni, Emanuele Bury, Erika Buratti, Rino Froldi, Marta Cippitelli, Gianmario Mietti, Mariano Cingolani

**Affiliations:** Forensic Medicine Laboratory, Institute of Legal Medicine, University of Macerata, 62100 Macerata, Italy; emanuelebury92@gmail.com (E.B.); burattierika@gmail.com (E.B.); rino.froldi@unimc.it (R.F.); cippitelli.marta@gmail.com (M.C.); gianmario.mietti@gmail.com (G.M.); mariano.cingolani@unimc.it (M.C.)

**Keywords:** morphine, opioids, fingernails, immunohistochemistry, ultra-high-performance liquid chromatography, high-resolution mass spectrometry, forensic toxicology, unconventional matrices

## Abstract

This study aimed to investigate the detection of morphine in fingernails from forensic autopsies using immunohistochemistry (IHC), with confirmation by ultra-high-performance liquid chromatography coupled with high-resolution mass spectrometry (UHPLC-HRMS). A primary antibody specific to morphine and a secondary antibody conjugated to horseradish peroxidase (HRP) was used. IHC on specimens of Subjects A and B (both drug addicts) resulted in the detection of morphine on a cell layer of the nail plate matrix. UHPLC-HRMS and GC-MS analysis showed that Subject A had a morphine concentration of 0.35 ng/mg in the fingernail and 472 ng/mL in the blood, while Subject B reached 1.23 ng/mg in the fingernail and 360 ng/ml in the blood. Most of those matrices were positive for codeine, methadone, EDDP, and 6-MAM. The use of IHC in Subject C (a former addict) showed no positivity for morphine in the fingernail, while the UHPLC-HRMS analysis confirmed its absence in the fingernail and blood. Additionally, an analysis of the scalp or pubic hair of the subjects was carried out using UHPLC-HRMS. The results suggest that IHC can be used to establish the site of accumulation of morphine in the nail matrix; for postmortem diagnosis; and that basic substances can be detected by UHPLC-HRMS. There are no previous studies on the use of IHC as a technique for forensic purposes in unconventional matrices, such as nails.

## 1. Introduction

Nails are made of keratin and fingernails grow at an average rate of 3 mm per month [1]. Unconventional matrices, such as nails, can provide important samples for clinical and forensic toxicology in the postmortem detection of drugs, but the mechanism of drug incorporation into nails is still unclear.

Studies suggest that drugs are primarily incorporated into nails by deposition into the matrix via the blood flow; other studies have demonstrated that certain drugs are incorporated via the nail bed [2]. In addition to the intake of drugs, exposure to environmental contamination and biological fluids are possible mechanisms of drug incorporation into nails, as is the case with the hair matrix [3]. The stability of drugs in nails makes their analysis very useful for postmortem investigations, especially when it is difficult to perform other tests (using other matrices) or when the material is too decomposed to produce reliable results.

Immunohistochemistry (IHC) is a technique that makes use of antigen-antibody binding to localize specific antigens in cells and tissue. IHC is not frequently used in forensic pathology. It has been applied in previous research to analyze forensic samples [4] or to study the morphine distribution in poisoning cases [5], using organs as the principal matrix. However, no study to date has applied this technique using unconventional matrices like nails. The nails, hair, and blood samples used for this study were obtained from autopsies of subjects with a suspected addiction to opioids.

## 2. Materials and Methods

### 2.1. Materials

#### 2.1.1. Matrices

Analyses were performed on fingernail samples from the forensic autopsies of two drug addicts (Subjects A and B) and a former addict (Subject C). The entire index fingernail was removed postmortem, including the matrix portion. Subsequently, the fingernail of the same subject was cut in half so that two equal portions of the same sample could be used for both analytical methods. All three subjects were men. Subject A was 44 years old, Subject B was 57 years old, and Subject C was 38 years old.

#### 2.1.2. Antibodies

Primary Antibody: Polyclonal anti-Morphine Antibody produced in Sheep (ARG23594, Arigo Biolaboratories, Hsinchu City 300 Taiwan, ROC).

Secondary Antibody: Monoclonal Anti-Goat/Sheep IgG-Peroxidase Antibody produced in Mouse (A9452, Sigma-Aldrich, Burlington, MA, USA).

#### 2.1.3. Reagents for Immunohistochemistry

PBS: NaCl (Carlo Erba, Cornaredo, Italy), NaH_2_PO_4_·H_2_O (J.T.Baker, Phillipsburg, NJ, USA), and NaH_2_PO_4_·2H_2_O (Carlo Erba, Cornaredo, Italy).

3,3 Diamenobenzidine tablets (Sigma-Aldrich, Burlington, MA, USA), Bovine Serum Albumin (Sigma-Aldrich, Burlington, MA, USA), Gelatine (Carlo Erba, Cornaredo, Italy), Mayers Hematoxylin (Bio-Optica, Milano, Italy), Saponins (Carlo Erba, Cornaredo, Italy), and Triton X-100 (Carlo Erba, Cornaredo, Italy) were used. 

#### 2.1.4. Chemicals and Reagents for UHPLC Analysis

Nalorphine (internal standard (IS) for the analysis in GC-MS), proadifen (SKF) (internal standard (IS) for the analysis in UHPLC), and MSTFA (N-methyl-N-(trimethylsilyl)-trifluoroacetamide) were purchased from Sigma.

Standards of morphine, 6-monoacetylmorphine, methadone, EDDP, and codeine were purchased from Sigma. Methanol (MeOH) for analysis, water for analysis, dichloromethane, 2-propanol, ammonium hydroxide of reagent grade, methanol for HPLC, and ultrapure water for HPLC were obtained from Carlo Erba; Isolute HCX (130 ng/10 mL) from Biotage. All reagents were of analytical grade and stored according to the manufacturer’s instructions.

### 2.2. Methods

#### 2.2.1. Immunohistochemistry

##### Paraffin Embedding

Tissues were not fixed in formaldehyde to prevent the extraction of basic substances in the fixative fluid. Tissue dehydration was performed by incubation in solutions of increasing concentration of ethanol and left in xylene overnight. Tissues were transferred to liquid paraffin at 60 °C and allowed to cool. From the tissue blocks, sections of 2 um were cut on a rotating microtome then collected on SuperFrost Plus slides (Menzel Gläser) and left to dry at 60 °C for one hour.

Sections were deparaffinized with xylene for 20 min and then passed through 3 cycles of 10 min in 99% ethanol and 2 cycles of 10 min in 96% ethanol. Endogenous peroxidase activity was then blocked with 0.35% H_2_O_2_ in methanol for 30 min. Rehydration was completed by rinsing in 96% ethanol, 10 min in 70% ethanol, and triple rinsing in distilled water.

##### Antibody and Peroxidase Marking

The sections were washed three times in a blocking solution containing 10 mM PBS with 1% BSA, 0.2% gelatin, and 0.05% saponin. The antibodies were diluted in 10 mM PBS containing 0.1% BSA and 0.3% Triton X-100, and the sections were incubated with the primary antibodies for 1 h at room temperature and then at 4 °C overnight. The next day, once they reached room temperature, the sections were triple-rinsed in PBS containing 0.1% BSA, 0.2% gelatin, and 0.05% saponin, and incubated with secondary antibodies diluted in PBS with 0.1% BSA and 0.3% Triton X-100 for 1 h. After an additional 3 washes in a solution of PBS with 0.1% BSA, 0.2% gelatin, and 0.05% saponin, peroxidase activity was detected after 10 min of incubation using diaminobenzidine (3.3 Diamenobenzidine tablets, Sigma-Aldrich) at a concentration of 1 mg/mL. DAB tablets were thawed approximately 20 min before use and activated with 0.35% H_2_O_2_ just before use. Then, the sections were rinsed in PBS for 3 cycles of 10 min, twice in distilled water, and afterward were counterstained in Mayers Hematoxylin for 2 min and placed under cold running water for 20 min. Dehydration was performed by incubation for two 3 min cycles in 70%, 96%, and 99% ethanol, followed by three 5 min cycles in xylene before coverslips were finally mounted using Eukitt (ORSatec). The immunohistochemical protocol is inspired by the one developed by Paulsen, I.M et al. [6]. The semiquantitative evaluation of the immunohistochemical reaction was performed by expert histologists using the Nikon Eclipse E200 light microscope. The intensity of the DAB signal was measured using the free software ImageJ Fiji, as described by Crowe et al. [7].

#### 2.2.2. Sample Preparation and Extraction

##### Extraction Procedure in Nail and Hair Matrices

The extraction procedure, specific to morphine, was carried out according to previous work [8]. Half of the nail was used for immunohistochemical analysis, the other half was used for UHPLC analysis (about 60 mg). Both the nail and hair (scalp or pubic) was washed with water, dried, and cut into small pieces.

The sample was extracted by adding 490 µL of distilled water with 0.1% of formic acid and 10 µL of methanol with 0.1% of formic acid. 20 ng of IS (SKF) were added. Hair samples were incubated for 24 h at 55 °C, while nail samples were incubated for 72 h at 55 °C. After incubation, the samples were centrifuged at 12.293× *g* in an ultracentrifuge. The supernatant was collected and evaporated; subsequently, the samples were resuspended with 50 µL of phase B (Methanol + 1% formic acid) for chromatographic injection.

##### Acid Hydrolysis and Extraction Procedure in Blood Matrix

The acid hydrolysis and extraction procedure were carried out according to previous work [9]. Morphine is found in the blood as 3-glucuronide and 6-glucuronide forms after administration, so it was extracted by acid hydrolysis, detaching glucuronides to reveal the total amount of free morphine [10]. A total of 2 mL of blood was added with 200 µL of chloridric acid and 250 ng of IS. The samples were incubated for 24 h at 55 °C.

The solid-phase extraction procedure followed the method used in our laboratory described in previous work [11].

##### Derivatization

The eluted samples were completely evaporated and then derivatized with 20 µL of MSTFA at 60 °C for 20 min. One microliter of the derivatized sample was injected into the GC-MS.

#### 2.2.3. Hr-LC and GC-MS Parameters

##### Hair and Nail Analysis and Quantification by UHPLC-HRMS

The Thermo Scientific Dionex Ultimate 3000 chromatographic system (UHPLC) coupled with Thermo Exactive Plus Orbitrap (HR-MS) was used for hair and nail analysis. The conditions applied for chromatographic analysis were as follows: the column used was Kinetex Biphenyl 2.6 µm (50 × 2.1 mm) by Phenomenex; column flow was set at 0.4 mL/min. Phase A used H_2_O + 0.1% formic acid; Phase B used MeOH + 0.1% formic acid. The column temperature was set to 25 °C. The elution gradient is shown in Table 1.

Scheme 60: For the identification of analytes, exact mass (EM) obtained from In Source Collision Induced Dissociation (50 eV) (In source CID), with an acceptance range of ±5 ppm, and production (PI) were used. The values monitored for analytes were as follows: 286.14377 (EM) (PI: 201.09101, 229.08592, 183.08044) for morphine; 328.15433 (EM) (PI: 211.07540, 183.08040, 193.06480) for 6-MAM; 300.15942 (EM) (PI 215.10666, 243.10157, 199.07536) for codeine; 310.21654 (EM) (PI: 105.03349, 219.11683, 195.11683) for methadone; 287.19033 (EM) (PI: 234.12773, 249.15120, 186.12773) for EDDP; and 354.24276 (EM) (PI: 167.08553, 91.05423, 105.06988) for SKF (IS).

##### Blood Analysis and Quantification by GC-MS

The GC-MS Polaris-Q was used for blood analysis. Analytical conditions were as follows: a capillary column (ZB 5 MS 30 m × 0.25 mm × 0.25 µm); helium as a carrier gas at a flow rate of 1.5 mL/min; a temperature program that started at 100 °C for 1 min and was increased first to 220 °C at 30 °C/min for 1 min, and then to 320 °C at 20 °C/min for 6 min (total run time of 17 min); an injection volume that was 1 µL in splitless mode; Full Scan mode, and a mass spectra range of 70–500. Subsequently, the specific SIM layout for morphine, 6-MAM, codeine, methadone, and EDDP was applied. The ion values monitored for analytes were as follows: m/z 429 414 324 for morphine-TMS, m/z 399 340 287 for 6-MAM-TMS, m/z 371 178 196 for codeine-TMS, m/z 72 294 223 for methadone, m/z 276 277 262 for EDDP, and m/z 455 414 440 324 for nalorphine-TMS (IS).

#### 2.2.4. Validation

The method was validated for linearity, quantitation limits (limit of detection (LOD) and limit of quantitation (LOQ)), according to the Scientific Working Group for Forensic Toxicology guidelines (SWCTOX) [12].

Standard curves for morphine and 6-MAM were obtained from previously checked blank samples (hair, nails, and blood) spiked with six concentration points with three replicates for each. Concentrations ranging from 0.05 to 5 ng/mg were prepared for hair and nail HR-LC analysis and concentrations ranging from 0.2 to 10 ng/mg were prepared for blood GC-MS analysis.

The area (analyte)/area (IS) was plotted against the known concentrations of the standard solutions to establish calibration equations. A linear regression equation was calculated using the least-squares method. LOD was determined according to the standard deviation of y-intercepts and the average slope of regression lines [(3.3∙s_y_)/Avg_m_]. The LOQ value was the lowest concentration showing acceptable values of bias (±20%) and precision (CV ≤ 20%); for details, see Table 2.

## 3. Results

**Figure 1:**
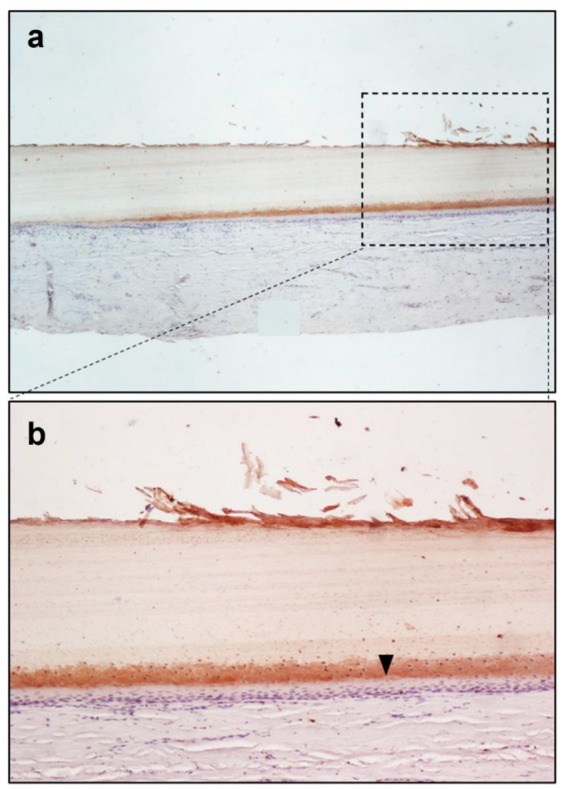
Immunoperoxidase staining for Sheep Anti-Morphine in the fingernail of Subject A. FigPicture (**a**) shows the 4× magnification, while picture (**b**) shows the 10× magnification. The figure shows the cytoplasmic staining of nail matrix cells of Subject A, indicating morphine positivity, shown in particular by a black arrow on the 10× picture.

**Figure 2:**
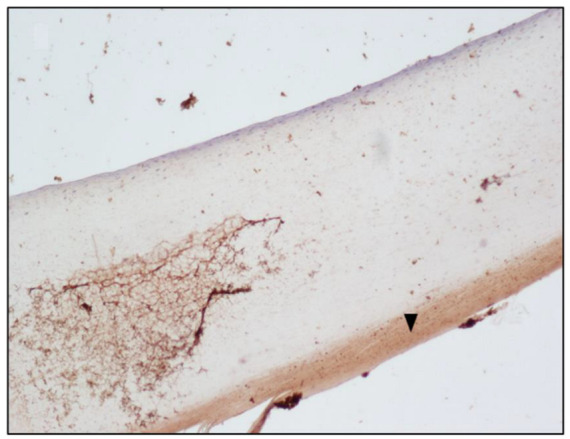
Immunoperoxidase staining for Sheep Anti-Morphine in the fingernail of Subject B, 10× magnification; the picture shows the peroxidase reaction across the nail matrix cells of Subject B, indicating morphine positivity, shown in particular by a black arrow.

The semiquantitative evaluation of the immunohistochemical reaction, performed by expert histologists using the Nikon Eclipse E200 light microscope, suggested an accumulation of morphine on the cytoplasm of the epithelial cells, at the level of the nail plate matrix, located in the germinal matrix section of the nail in drug-addicted subjects (Figure 1 and Figure 2). “Subject A” and “Subject B” were drug addicts, while “Subject C” was a former addict.

**Figure 3:**
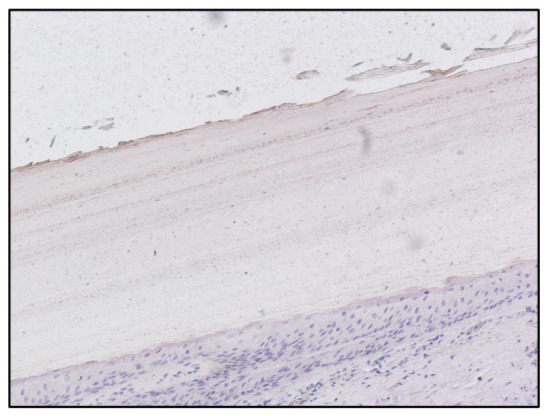
Immunoperoxidase staining for Sheep Anti-Morphine in the fingernail of Subject A, 10× magnification. The immunohistochemistry protocol has been modified to observe the presence of any non-specific reaction; in this figure the primary antibody has been used, while the secondary antibody conjugated to the peroxidase enzyme has not been used.

**Figure 4:**
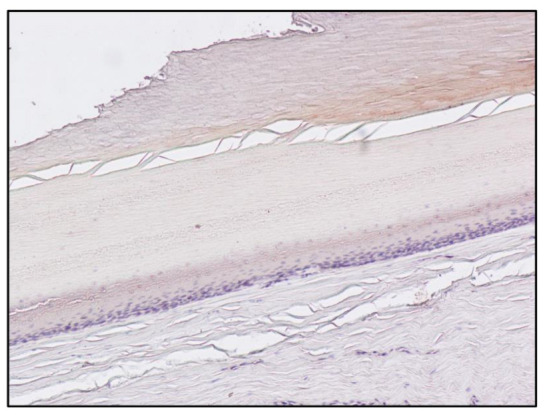
Immunoperoxidase staining for Sheep Anti-Morphine in the fingernail of Subject A, 10× magnification. The immunohistochemistry protocol has been modified to observe the presence of any non-specific reaction; in this figure the primary antibody has not been used, while the secondary antibody conjugated to the peroxidase enzyme has been used.

**Figure 5:**
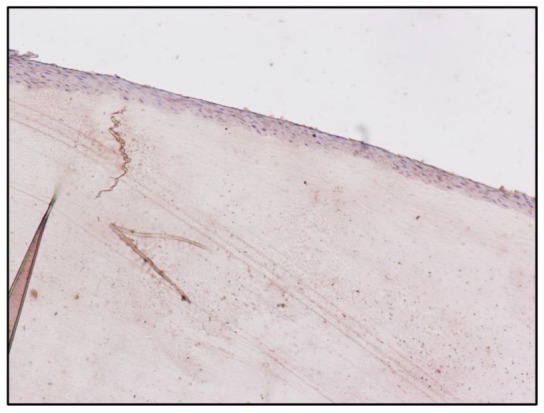
Immunoperoxidase staining for Sheep Anti-Morphine in the fingernail of Subject C, 10× magnification. The pictures show an absence of positivity for morphine in the nail matrix cells. Counterstaining with hematoxylin. Primary Ab: Polyclonal anti-Morphine Antibody produced in Sheep, dil. 1:100. Secondary Ab: Monoclonal Anti-Goat/Sheep IgG-Peroxidase Antibody produced in Mouse, dil. 1:100, original magnification: 10×.

**Figure 6:**
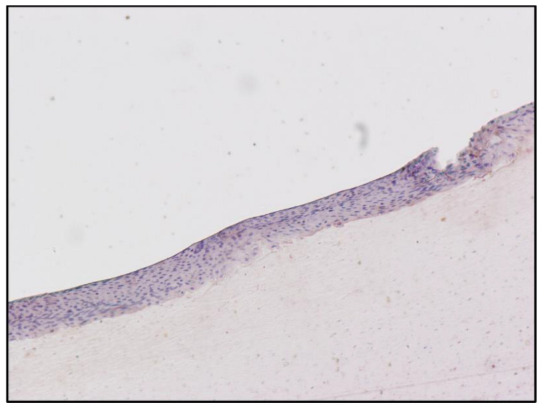
Immunoperoxidase staining for Sheep Anti-Morphine in the fingernail of Subject C, 10× magnification. The pictures show an absence of positivity for morphine in the nail matrix cells. Counterstaining with hematoxylin. Primary Ab: Polyclonal anti-Morphine Antibody produced in Sheep, dil. 1:100. Secondary Ab: Monoclonal Anti-Goat/Sheep IgG-Peroxidase Antibody produced in Mouse, dil. 1:100, original magnification: 10×.

The no-primary antibody and no-secondary antibody controls were performed to determine if those antibodies were binding non-specifically to cellular components that did not contain the biomarker or protein of interest. It was applied to the fingernail of Subject A and was successful, showing no staining at all (Figure 3 and Figure 4). Both antibodies were used on the fingernail of the former addict (Subject C), as a negative control, with no reaction detected in any cell of the tissue (Figure 5 and Figure 6). The results of the DAB intensity measure using ImageJ Fiji are shown in Table 3, where “Area” gives the size of the IHC image, and “Mean grey value” represents the quantified signal. These data confirm the positive staining given by the peroxidase to the fingernail of the drug-addicted subjects and its absence in the formerly addicted subject.

The primary antibody is specific for morphine and exhibits negligible cross-reaction with codeine. Over the years, numerous works have been carried out to find an antibody with no cross-reaction with other opioids [13]. Even if cross-reactions do not seem to affect the reliability of the results, UHPLC confirmation is important to be able to classify the similar molecules found in the sample, morphine, and codeine in this case. UHPLC analysis was able to distinguish several substances and their respective metabolites, as shown in Table 4. The quantitative analysis of morphine in Subject A showed a morphine concentration of 0.35 ng/mg in the fingernail, 3.64 ng/mg in scalp hair, and 472 ng/mL in blood, while 6-MAM concentrations were 0.43 ng/mg in the fingernail, 1.42 ng/mg in scalp hair, and negative in blood. The morphine in Subject B reached 1.23 ng/mg in the fingernail, 1.60 ng/mg in pubic hair, and 360 ng/mL in blood, while 6-MAM concentrations were 1.18 ng/mg in the fingernail, 0.44 ng/mg in pubic hair, and negative in blood. All these matrices were positive for codeine, methadone, and EDDP in both subjects. The use of immunohistochemistry in the case of the former addict (Subject C) led us to infer an absence of positivity for morphine in the fingernail; the UHPLC-HRMS analysis confirmed its absence in the fingernail and blood. On the other hand, the fingernail of this subject presented 1.03 ng/mg of 6-MAM, while the concentration of morphine in the pubic hair matrix was 2.2 ng/mg, with 4.43 ng/mg of 6-MAM and codeine, methadone, and EDDP positivity.

## 4. Discussion

In the field of forensic toxicology, it is well-known that the detection of drugs in the blood is the most relevant way of determining the cause of death, given short-term information related to drug addiction. Long-term drug history can be traced by hair analysis [14,15]. A scalp hair matrix can be used to derive a chronological history of drug use, with an extended detection window of approximately 1 month per half-inch of hair, while pubic hair grows more slowly [16,17]. On the other hand, studies suggest that pubic hair can offer an alternative way of proving previous drug use, but it should be avoided when estimating drug use history, and higher quantitative results in pubic hair do not represent heavier drug use [18]. Furthermore, pubic hair does not grow continually like scalp hair; it has a longer resting phase and a different anagen to telogen ratio [19]. This could explain why the accumulation of substances in pubic hair can be higher than in scalp hair.

Over the past few decades, nails (fingernails and toenails) have become a useful specimen type for the detection of drug use and abuse [20]. The innovation of our study was not only to have introduced a new technique for identifying morphine in nails with an accurate stratification of substance accumulation but also to have made a comparison with other biological matrices, particularly hair and pubic hair as well as blood.

Regarding the results obtained from our samples, the UHPLC-HRMS analysis for Subjects B and C showed the presence of morphine and its metabolite in pubic hair. We believe that even if the fingernail has a keratinous matrix similar to hair, pubic hair shows a more long-term and more contaminated drug accumulation in comparison to the nail. The presence of 6-MAM in the fingernail sample of Subject C, analyzed by UHPLC-HRMS, demonstrates that there was no cross-reaction between 6-MAM and morphine during the immunohistochemical analysis.

Histologically, the nail matrix is composed of a thick stratified squamous epithelium that lacks a granular layer. Matrix cells divide, move distally, and cornify, forming the nail plate that slides over the nail bed. When forming the nail plate, matrical keratinocytes flatten and lose their nuclei; this occurs in the eosinophilic keratogenous zone. Below the keratogenous zone is the prekeratogenous zone and below that lies the basal layer. The prekeratogenous zone is made of polygonal cells with clear cytoplasm and oval nuclei arranged parallel to the nail plate and the reaction to the morphine antigen is located in the cytoplasm of those cells [21]. The nail root produces most of the volume of the nail plate and the nail bed. It can be assumed that morphine is transported by blood vessels and deposed in the cells of the nail germinal matrix. As the nail grows, those cells slide distally toward the nail-free margin, pushed by the newer cells of the nail matrix. Proceeding in this direction, the peroxidase reaction stops in the proximity of the nail bed, which extends from the edge of the nail root to the hyponychium [22].

Regarding the distribution of morphine, based on our results, we can assume that the drug is less incorporated via the nail bed and that the main mechanism of distribution is deposition into the nail matrix cells, transported by the blood flow.

The staining located in the cytoplasm of not yet keratinized cells can lead to speculations about the drug metabolism; its accumulation is probably correlated to its binding with other molecules like proteins and phospholipids. A hair matrix, composed of dead keratinized cells, could be the right comparison to better understand the exact location of where the drugs can be incorporated and accumulated [23]. For instance, drugs such as nicotine, morphine, cocaine, and amphetamine are weak bases and they bind to the melanin with electrostatic bonding, because it is acidic [24]. The dead keratinized cells of the hair shaft medulla contain a high level of melanin. On the other hand, in the proximal nail matrix, melanocytes typically lie dormant where the nail originates, leading to a lower level of melanin production in the nail plate compared to the hair shaft [25]. Further studies are needed to better understand the kinetics of weak bases such as morphine inside the nail cells and hair shaft.

The absence of peroxidase reaction in the narrow-keratinized cells of the nail plate could be explained by the differential staining properties of the nail plate [26,27,28,29].

The search for exogenous substances on nail material is an ongoing subject of study and more research is required to better understand its characteristics. Further studies are needed to determine if the period of substance intake can influence the specific location of the morphine-accumulating cell layer in the nail matrix or on the nail bed; this could potentially be used as a method to study the addiction history of heroin addicts.

## 5. Conclusions

The detection of a drug of abuse in the nails indicates an intake in an antecedent period, which can vary from a few weeks up to several months. This matrix certainly represents a new frontier of research in the medico-legal field and, above all, in the forensic toxicology field. There is a need to carry out studies aimed at defining the temporal determination of the intake, due to the double path of blood flow to the nails from the root and the bed, and due to the double growth mechanism [30]. On the other hand, nails are still a valid alternative to hair, especially if we consider characteristics such as the absence of ethnic differences in the composition of the nail and the fact that it is more difficult to alter nail samples compared to hair.

Immunohistochemistry represents an innovative technique in the forensic toxicology field. This work demonstrates that immunohistochemical analysis can be applied for forensic purposes in unconventional matrices, such as nails, which can be successfully used to establish the site of accumulation of substances such as morphine, and for postmortem diagnosis in autopsy specimens of alternative matrices. This study aimed to enrich our scientific knowledge about the use of unconventional matrices and to investigate how a substance can accumulate in this kind of material. The research has also shown that substances such as morphine, 6-MAM, codeine, methadone, and EDDP can be detected in nails using UHPLC-HRMS, with the ability to distinguish changes in intake over time, based on the distribution of the substance in the matrix and on quantitative assessments.

## Figures and Tables

**Table 1:** UHPLC elution gradient.

TIME	PHASE A (%)	PHASE B (%)
0–0.5	98	2
0.5–10	0	100
10–12	0	100
12–13	98	2
13–15	98	2

**Table 2:** Summary of validation results.

Hair			
Substances	Linearity Range	R2	LOQ(LOD)
Morphine	0.05–5 (ng/mg)	0.9933	0.05 (0.02) (ng/mg)
MAM	0.05–5 (ng/mg)	0.9897	0.05 (0.02) (ng/mg)
**Nail**			
Morphine	0.05–5(ng/mg)	0.9942	0.05 (0.02) (ng/mg)
MAM	0.05–5 (ng/mg)	0.9853	0.05 (0.02) (ng/mg)
**Blood**			
Morphine	0.5–500 (ng/mL)	0.9959	0.5 (0.2) (ng/mL)

**Table 3:** ImageJ Fiji DAB intensity measure.

Fingernail matrix	Area	Mean
Subject A (Figure 1)	1,228,800	19.695
Subject B (Figure 2)	1,228,800	26.049
Subject C (Figure 5)	1,228,800	2.252

**Table 4:** UHPLC and GC-MS analysis results for morphine and opioids concentrations.

**Subject A matrix**	**Instrument**	**Morphine**	**6-MAM**	**Codeine**	**Methadone**	**EDDP**
Blood	GC-MS	472 ng/ml	Negative	Positive	Positive	Positive
Scalp hair	UHPLC	3.64 ng/mg	1.42 ng/mg	Positive	Positive	Positive
Fingernail	UHPLC	0.35 ng/mg	0.43 ng/mg	Positive	Positive	Positive
**Subject B matrix**	**Instrument**	**Morphine**	**6-MAM**	**Codeine**	**Methadone**	**EDDP**
Blood	GC-MS	360 ng/ml	Negative	Positive	Positive	Positive
Pubic hair	UHPLC	1.60 ng/mg	0.44 ng/mg	Positive	Positive	Positive
Fingernail	UHPLC	1.23 ng/mg	1.18 ng/mg	Positive	Positive	Positive
**Subject C matrix**	**Instrument**	**Morphine**	**6-MAM**	**Codeine**	**Methadone**	**EDDP**
Blood	GC-MS	Negative	Negative	Negative	Negative	Negative
Pubic hair	UHPLC	2.2 ng/mg	4.43 ng/mg	Positive	Positive	Positive
Fingernail	UHPLC	Negative	1.03 ng/mg	Negative	Negative	Negative

## Data Availability

All data are contained in the manuscript.
